# Clinical and imaging characteristics of patients with bronchogenic cysts: a single-center retrospective analysis

**DOI:** 10.1186/s12880-023-01042-1

**Published:** 2023-09-14

**Authors:** Tan-tan Ma, Geng Chen, Dan Wang, Hong Xu, Jian-guang Zhang

**Affiliations:** Department of Gastroenterology, The First Hospital of Jilin University, 71 Xinmin Street Changchun, Jilin, 130021 China

**Keywords:** Bronchogenic cysts, Mediastinum, Imaging analysis, Thyroid cancer

## Abstract

**Background:**

Bronchogenic cysts (BCs) are rare and usually asymptomatic malformations detected during imaging examinations. We aimed to investigate the clinical and imaging characteristics of patients with BCs.

**Methods:**

We retrospectively evaluated patients who received surgery to remove their BCs from January 2015 to January 2019. Their baseline characteristics, clinical information, and imaging results were reviewed.

**Results:**

Our study included 129 patients, with 57 males and 72 females and a mean age of 42.7 years old. The most common location for BCs was the mediastinum (67 patients, 51.9%). Fewer than half of the patients (53 patients, 41.1%) reported clinical symptoms, with chest pain being the most common (16 patients, 30.2%). Neck BCs were more frequently observed in young patients (*P* = 0.002) and were more often associated with thyroid cancer (*P* = 0.007). A computed tomography scan was the most commonly used method to diagnose BCs in the lung and mediastinum, whereas ultrasound was the most commonly used diagnostic method for neck BCs. The characteristic images were well-defined, thin-wall cystic lesions in varying densities. A few lesions showed small, calcified spots along the rim or cavities.

**Conclusions:**

Although most BCs were found in the mediastinum, their locations could vary in different sex and age groups. Particular attention should be paid to young patients with BCs in the neck to rule out thyroid cancer.

## Background

Bronchogenic cysts (BCs) are rare congenital malformations derived from the endoderm of the developing respiratory system. They are lined by the respiratory-type pseudostratified ciliated columnar epithelium [1, 2]. The definitive pathogenesis of these cysts remains unclear. BCs are usually asymptomatic and can be observed at any age, from infancy to adulthood. Imaging is an effective way to detect the presence of BCs [3]. The most common location for BCs is the mediastinum, followed by the digestive tract, pericardium, and skin [2]. Rare locations, such as the spine, diaphragm, pancreas, saddle area, medulla, intramural esophagus, and thoracic wall, were also reported [1, 4–8].

Clinically, patients with BCs can have no symptoms. However, life-threatening illnesses can also happen, usually due to complications when the BCs enlarge [3, 9]. The enlarged BCs could lead to chest pain, cough, expectoration, hemoptysis, dyspnea, numbness, and/or weakness in the limbs or back. When the BCs are at a critical location, they could also compress adjacent organs and nerves to cause paralysis. Appropriate surgical resection is the treatment choice for these BCs. However, the accurate diagnosis of BCs relies on histopathological examination. Pre-operative identification of BCs based on radiological findings is challenging. There are no specific clinical or radiological criteria to diagnose BCs. The clinical and imaging characteristics of BCs are rarely reported.

Therefore, in the present study, we retrospectively evaluated patients with BCs treated at our hospital to describe the clinical and imaging characteristics of BCs to facilitate their management in clinical practice.

## Methods

### Study design and participant selection

We performed a retrospective study and reviewed patients hospitalized at the First Hospital of Jilin University in Jilin, China, between January 2015 and January 2019. The study protocol was approved by the hospital ethics committee. Due to the retrospective study design, informed consent was waived by the First Hospital of Jilin University. The inclusion criteria were patients with (1) surgical lesion resection with complete pre-operative and post-operative images; and (2) post-operative pathological diagnosis of the BCs [10]. Those patients with incomplete medical records or poor image quality were excluded.

### Data collections

Medical records were reviewed to collect information, including sex, age, clinical symptoms, complications (airway compression, infection, and abscess), thyroid cancer, and imaging characteristics.

### Image analysis

All imaging data, including computed tomography (CT), magnetic resonance imaging (MRI), and Doppler ultrasonography, were documented. The results were retrieved and analyzed if the patient received a bronchoscopy or endoscopic ultrasonography (EUS) examination. Two radiologists with at least ten years of experience evaluated all imaging data separately. A consensus was reached for the final characteristic determinations.

### Statistical analysis

The R statistical software (version 4.2.2, https://www.r-project.org/) was used for the statistical analyses. The continuous data are presented as mean standard deviation and compared by the Student t-test, or median with interquartile ranges and compared by the non-parametric test, when appropriate. The categorical data are presented as numbers with percentages and were compared by the Chi-square test. A *P <* 0.05 was considered statistically significant.

## Results

### Participant characteristics

**Fig. 1:**
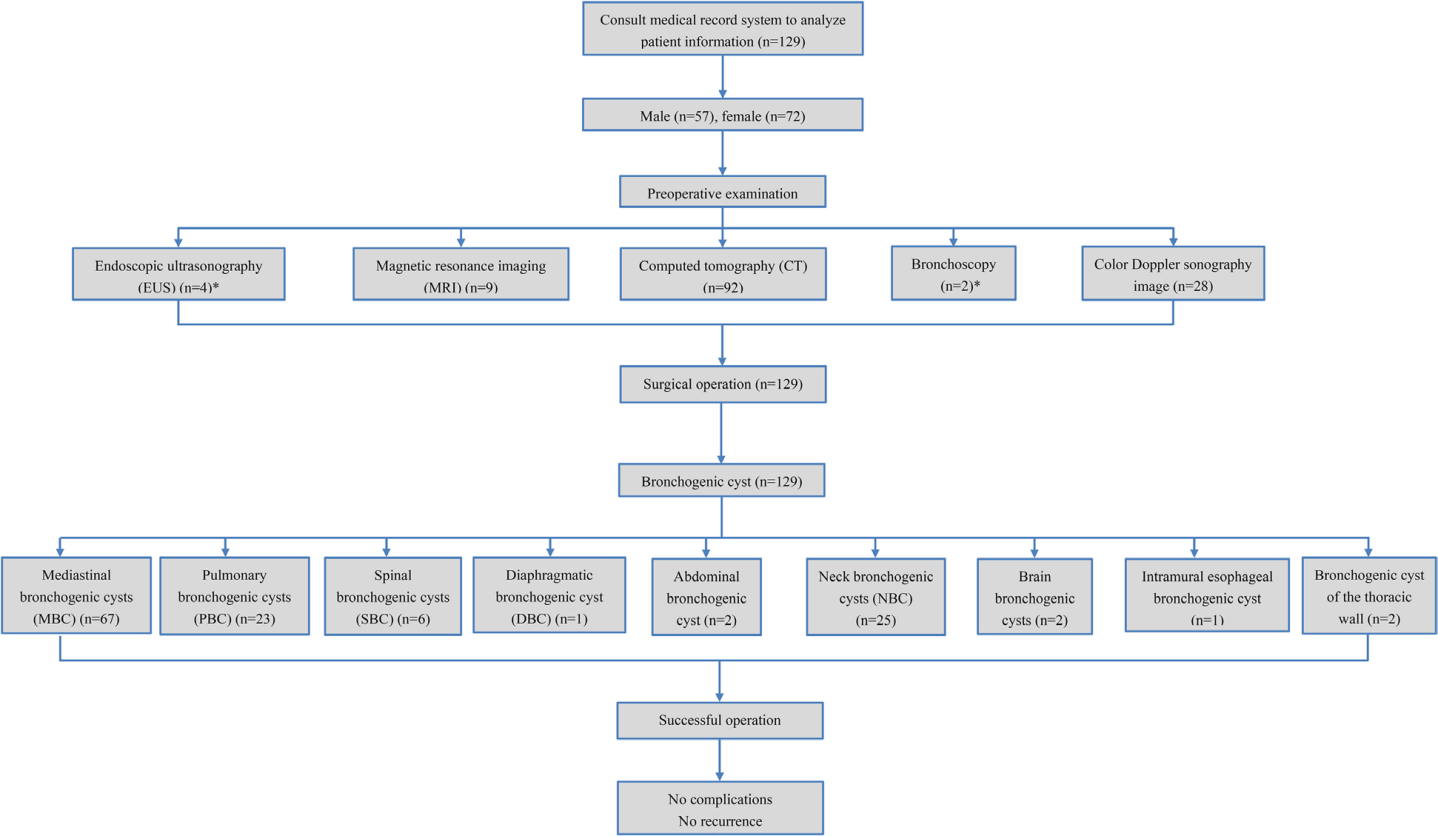
Flow chart of patient selection * Patients who received the EUS also received the CT scan (n = 4). Patients who received the bronchoscopy also received the CT scan (n = 2)

One hundred and twenty-nine BC patients were enrolled in this retrospective study (57 males and 72 females) (Fig. 1). The age of these patients ranged from 5 months to 69 years, with a mean age of 42.7 years.

### Clinical findings

As shown in Table 1, the mediastinum was the most common location for the BCs (67 patients, 51.9%). Fewer than half of the patients (53, 41.1%) reported clinical symptoms. The most common symptoms were chest pain (16 patients, 30.2%) and cough (13 patients, 24.5%). We further compared the characteristics of patients with BCs in different locations (Table 2). Most BCs in males were located in the lungs (*P* = 0.008). Patients with BCs in the neck were younger than patients with BCs in other locations (*P* = 0.002). The BCs in the neck or locations other than the mediastinum and lungs were more likely to be associated with thyroid cancer (*P* = 0.007), whereas the BCs in the lungs were more likely to have a concurrent infection (*P* = 0.003).

**Table 1:** Location, symptoms, and pre-operative examinations of the bronchogenic cysts

Characteristics	Patients N (%)
Location	
Mediastinal bronchogenic cysts	67 (51.9)
Pulmonary bronchogenic cysts	23 (17.8)
Spinal bronchogenic cysts	6 (4.7)
Diaphragmatic bronchogenic cyst	1 (0.8)
Abdominal bronchogenic cyst	2 (1.6)
Neck bronchogenic cysts	25 (19.4)
Brain bronchogenic cysts	2 (1.5)
Intramural esophageal bronchogenic cyst	1 (0.8)
Bronchogenic cyst of the thoracic wall	2 (1.5)
Symptoms	
Chest pain	16 (30.2)
Cough	13 (24.5)
Chest distress	9 (17.0)
Hemoptysis	2 (3.8)
Dyspnea	2 (3.8)
Fever	1 (1.9)
Headache	1 (1.9)
Limb paralysis and/or aphasia	6 (11.2)
Hoarseness	1 (1.9)
Eating choking sensation	1 (1.9)
Memory declines	1 (1.9)
Pre-operative examination	
Computed tomography	92 (71.3)
Magnetic resonance imaging	9 (7.0)
Ultrasound	28 (21.7)

**Table 2:** Comparison of the characteristics of patients with bronchial cysts in different locations

Characteristics	Location, N(%)				*P*
	Mediastinum	Lungs	Neck	Others	
Male	31(46.3%)	16(69.6%)	7(28.0%)	3(21.4%)	0.008
Age, years, median (IQR)	52.0(13.8)	49.5(7.3)	16.0(44.8)	42.0(17.0)	0.001
Complications	16(23.9%)	7(30.4%)	9(36.0%)	4(28.6%)	0.699
Thyroid cancer	0(0.0%)	2(8.7%)	4(16.0%)	3(15.0%)	0.007
Infection	0(0.0%)	3(13.0%)	0(0.0%)	0(0.0%)	0.003
Hypertension	6(9.0%)	1(4.3%)	1(4.0%)	0(0.0%)	0.543
Diabetes	6(9.0%)	1(4.3%)	0(0.0%)	0(0.0%)	0.271
Coronary artery disease	5(7.5%)	0(0.0%)	1(4.0%)	0(0.0%)	0.388
Pneumonia	4(6.0%)	1(4.3%)	0(0.0%)	0(0.0%)	0.498
Fistula	0(0.0%)	0(0.0%)	1(4.0%)	1(7.1%)	0.153
Diagnostic methods					
CT	67(100.0%)	20(87.0%)	2(8.0%)	3(21.4%)	< 0.001
MRI	0(0.0%)	0(0.0%)	1(4.0%)	8(58.1%)	< 0.001
Ultrasound	0(0.0%)	3(13.0%)	22(88.0%)	3(21.4%)	< 0.001

IQR, interquartile range

### Imaging characteristics

**Fig. 2:**
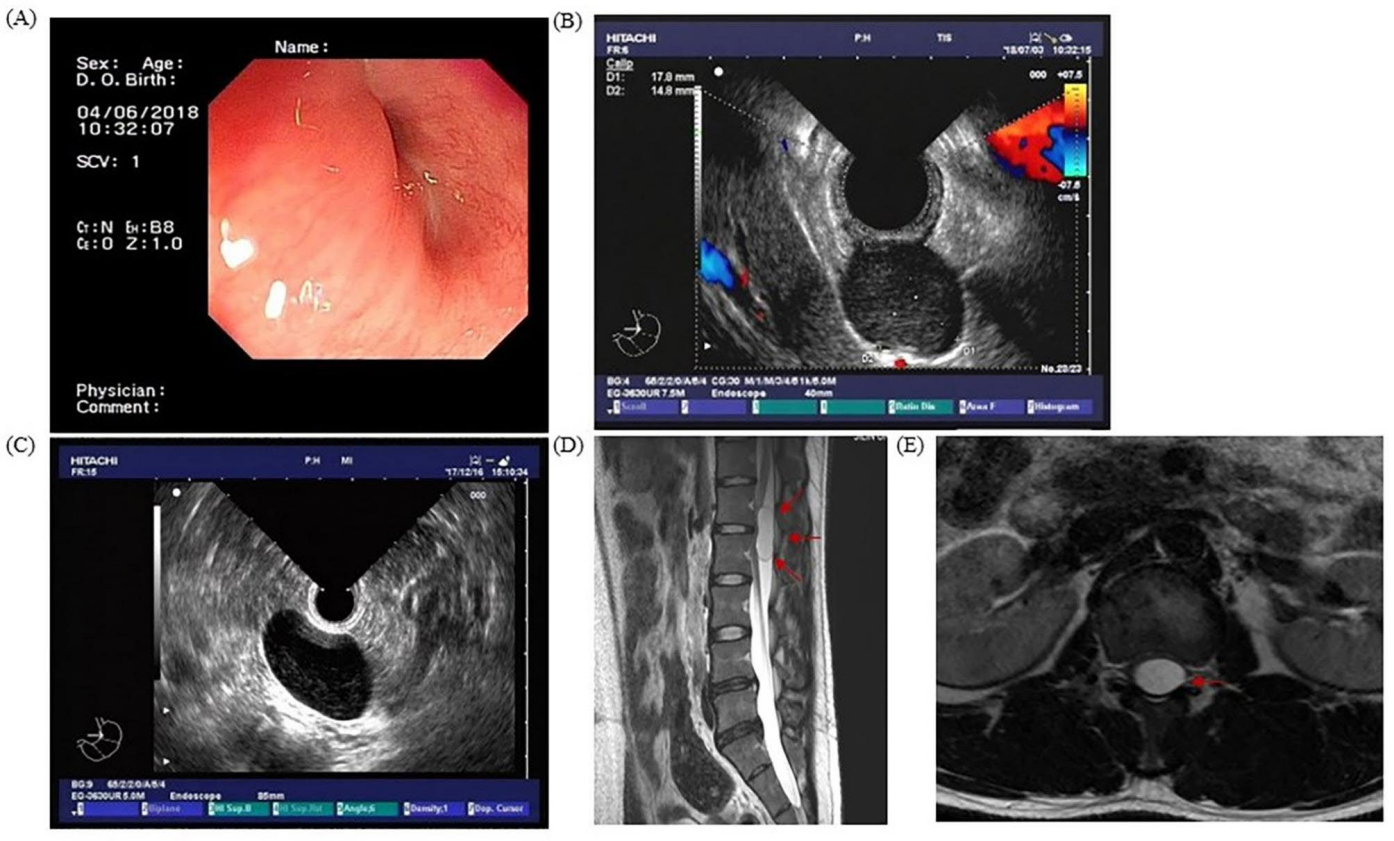
Representative images of patients diagnosed with BCs. (**A**, **B**), Case No. 1 with the post-operative diagnosis of a diaphragmatic bronchogenic cyst. Pre-operative endoscopic examination revealed a slight mucosal bulge in the lower esophagus near the dentate line (**A**). A well-defined oval hypoechoic mass with homogeneous internal echoes outside the wall of the bulging esophagus (13.9*19.6 mm in cross-section) was detected via the subsequent ultrasound microprobe examination and was suspected to be attached to the intrinsic muscular layer of the esophagus (**B**); (**C**), Case No. 2 with a post-operative diagnosis of a retroperitoneal bronchogenic cyst. Pre-operative ultrasound endoscopy found a smooth cystic mass outside the gastric wall without an echogenic signal (29.9 * 38.8 mm in cross-section); (**D**, **E**) Case No. 3 with a post-operative diagnosis of an intraspinal bronchogenic cyst. Pre-operative magnetic resonance imaging of the lumbar spine showed a cystic occupying lesion in the spinal canal near the T1 and T2 vertebrae (red arrows)

The diagnostic methods for bronchial cysts varied depending on their locations (Table 3). There were significant differences in the diagnostic methods for the BCs in different locations (*P <* 0.001). CT was more frequently used to diagnose the BCs in the mediastinum, lungs, and thymus, whereas ultrasound was mainly used to diagnose the BCs in the neck, chest wall, thyroid, and tracheal areas. MRI was commonly used to diagnose the BCs in the central nervous system, including the spinal canal, vertebral body, sacral region, and medulla oblongata. Regarding imaging characteristics of the BCs, pre-operative CT, MRI, and ultrasound were performed on 92, 9, and 28 patients, respectively. The CT images for 31 patients showed well-defined, thin-wall cystic lesions with varying densities. Analysis of these 31 lesions further demonstrated that 23 lesions mimicked a thymoma, 5 lesions showed several small, calcified spots along the rim of the cyst, and 3 lesions showed cystic cavities (Table 4). Four patients underwent the EUS examinations. The BCs in two patients mimicked esophageal leiomyoma. Three patients were found to have airway compressions during the bronchoscopic evaluations. Head and vertebral MRI demonstrated variable signal intensities on T1-weighted images and bright signal intensities on T2-weighted images. Neck color Doppler sonography images showed a primarily hypoechoic mass. Two patients had imaging characteristics indicating bleeding in the cyst. Representative examination images can be found in Fig. 2.

**Table 3:** Comparison of diagnostic methods for the bronchial cysts in different locations

Locations	Diagnostic mode			Total
	CT	MRI	Ultrasound	
Mediastinum				
Anterior	38(100.0%)	0(0.0%)	0(0.0%)	38(29.5%)
Middle	1(100.0%)	0(0.0%)	0(0.0%)	1(0.8%)
Mediastinum	11(100.0%)	0(0.0%)	0(0.0%)	11(8.5%)
Posterior	12(100.0%)	0(0.0%)	0(0.0%)	12(9.3%)
Thymus	4(100.0%)	0(0.0%)	0(0.0%)	4(3.1%)
Lungs				
Left superior lobe	3(100.0%)	0(0.0%)	0(0.0%)	3(2.3%)
Left inferior lobe	7(97.5%)	0(0.0%)	1(12.5%)	8(6.2%)
Right superior lobe	3(100.0%)	0(0.0%)	0(0.0%)	3(2.3%)
Right middle lobe	1(100.0%)	0(0.0%)	0(0.0%)	1(0.8%)
Right inferior lobe	5(100.0%)	0(0.0%)	0(0.0%)	5(3.9%)
Hilum	1(100.0%)	0(0.0%)	0(0.0%)	1(0.8%)
Parabronchus	1(100.0%)	0(0.0%)	0(0.0%)	1(0.8%)
Paratracheal	0(0.0%)	0(0.0%)	2(100.0%)	2(1.6%)
Neck	2(10.0%)	1(5.0%)	17(85.0%)	20(15.5%)
Thyroid gland	0(0.0%)	0(0.0%)	5(100.0%)	5(3.9%)
Chest wall	0(0.0%)	0(0.0%)	2(100.0%)	2(1.6%)
Saddle region	0(0.0%)	1(100.0%)	0(0.0%)	1(0.8%)
Central nervous system				
Spinal canal	0(0.0%)	5(100.0%)	0(0.0%)	5(3.9%)
Vertebral body	0(0.0%)	1(100.0%)	0(0.0%)	1(0.8%)
Medulla oblongata	0(0.0%)	1(100.0%)	0(0.0%)	1(0.8%)
Pancreas	1(100.0%)	0(0.0%)	0(0.0%)	1(0.8%)
Retroperitoneal	0(0.0%)	0(0.0%)	1(100.0%)	1(0.8%)
Diaphragmatic muscle	1(100.0%)	0(0.0%)	0(0.0%)	1(0.8%)
Ductal wall	1(100.0%)	0(0.0%)	0(0.0%)	1(0.8%)
Total	92(71.3%)	9(7.0%)	28(21.7%)	129(100.0%)

**Table 4:** Comparison of imaging characteristics of patients with bronchial cysts in different locations

Imaging characteristics	Location, N(%)				*P*
	Mediastinum	Lungs	Neck	Others	
Calcification	5(7.5%)	1(4.3%)	4(16.0%)	0(0.0%)	0.269
Thyroid nodule	0(0.0%)	2(8.7%)	9(36.0%)	0(0.0%)	< 0.001
Thyroid cancer	0(0.0%)	2(8.7%)	7(28.0%)	0(0.0%)	< 0.001
MRI T1 and T2 signal intensification	0(0.0%)	0(0.0%)	0(0.0%)	4(28.6%)	< 0.001
Thymoma-like	23(34.3%)	0(0.0%)	0(0.0%)	0(0.0%)	< 0.001
Cystic hemorrhage	0(0.0%)	1(4.3%)	1(4.0%)	0(0.0%)	0.329
Cystic cavity	0(0.0%)	3(13.0%)	0(0.0%)	0(0.0%)	0.003
Infection	1(1.5%)	1(4.3%)	1(4.0%)	0(0.0%)	0.738
Cyst	10(14.9%)	5(21.7%)	13(52.0%)	3(21.4%)	0.003
Anterior cyst fistula	0(0.0%)	0(0.0%)	3(12.0%)	1(7.1%)	0.017

## Discussion

This study analyzed the clinical and radiographic characteristics of 129 patients with BC. We found that the locations of BCs varied in different sex and age groups, and different locations of BC also had different radiographic features. Most BCs were found in the mediastinum. Neck BCs were more likely to be associated with thyroid cancer. Our study provides a better understanding of the clinical features and radiographic characteristics of patients with BCs.

BCs are rare congenital malformations derived from the foregut, with a prevalence of 1:42,000–1:68,000 [10]. Most patients with BCs were asymptomatic [11], and could cause clinical symptoms when they enlarged and compressed adjacent structures or had complications such as infection, perforation, or hemorrhage [12, 13]. In the present study, among 129 BC patients, 53 (41.1%) presented with symptoms, whereas 76 (58.9%) were asymptomatic. Unlike other studies that reported back or abdominal pain as the most common symptom [14], chest pain and cough were the predominant symptoms in our study, which might be attributed to the high percentage of mediastinal BCs in our study population. Additionally, young patients were more likely to have neck BCs, and 15% of neck BCs were associated with thyroid cancer. When we carefully reviewed the medical records, these BCs were discovered incidentally during surgery for thyroid cancer (nine cases) or were initially misdiagnosed as thyroid cancer (13 cases) [15, 16]. The association between neck BCs and thyroid cancer requires further studies. Meanwhile, clinicians who evaluate patients with neck BCs should rule out thyroid cancer. Clinicians who treat patients with thyroid cancer should also consider the possibility of BCs. Our findings provided a comprehensive description of the clinical and imaging characteristics of BCs, which could improve our understanding of the diagnosis and management of this rare illness.

Imaging studies play a significant role in diagnosing BCs and are commonly used to determine the nature of the lesions and decide whether further surgical treatment is necessary. CT scan is beneficial in the diagnosis of BCs in the bronchi [17]. The CT scan shows the cystic structures, with the density varying significantly based on the presence of highly proteinaceous, mucoid, bloody pigments, or calcium oxalate cystic contents. On the CT scans, BCs are usually sharply marginated cysts with soft-tissue or water attenuation. Most of them are cystic or cavity-like. Few of them show solid structures. In our study, imaging features consistent with thymoma were more likely to be observed in the mediastinal BCs. Calcification was relatively rare, but once it occurred, it could be challenging to distinguish from other diseases [18]. The appearance of MRI varied based on the content of the cyst, with variable signal intensities on T1 and bright signal intensities on the T2-weighted image, which was consistent with previous study reports [18, 19]. In the ultrasound images, BCs presented as well-circumscribed masses and hypoechoic tumors. Due to the complexity of imaging, surgical resection was recommended for diagnosis [20].

Considering that BCs are benign lesions and most patients are asymptomatic, a previous study recommended conservative observation as the treatment method for most BCs [21]. However, due to the increased complications and risk of malignant transformation in adulthood, 80% of the BCs in adult patients might be removed whether they show symptoms or are asymptomatic. Typical symptoms for patients with complications include bronchitis, pneumonia, pericarditis, sepsis, pain, dysphonia, hemoptysis, and dysphagia [19].

Surgical resection has been recommended for patients with BCs who have a risk for severe compilations [21]. Early surgical treatment can reduce morbidity and medical expenses, decrease post-operative recurrence, and minimize the risk of cyst abscesses and bleeding [13]. Early surgical resection could also reduce the possibility of malignant transformation [22, 23]. In the future, more studies are required to identify patients with BCs with characteristic clinical and imaging features to develop cancer [24, 25].

The strengths of our study were its large sample size and comprehensive evaluations of clinical and imaging characteristics. Most existing literature on BCs were case reports or studies with limited sample sizes. The limitations of our study included its single-center research with no long-term follow-up on patient outcomes. The retrospective study design could also bring biases to our results. More studies, especially studies from other geographic areas or different ethnic groups, are required to validate our study findings externally.

## Conclusion

Although most BCs were found in the mediastinum, their locations could vary in different sex and age groups. CT was commonly used to diagnose the BCs in the thoracic area, and ultrasound was used to diagnose neck BCs. Particular attention should be paid to young patients with BCs in the neck to rule out thyroid cancer.

## Data Availability

The datasets generated and analyzed during the present study are available from the corresponding authors upon reasonable request.

## List of Abbreviations

BCs: Bronchogenic cysts
CT: Computed Tomography
MRI: Magnetic Resonance Imaging
EUS: Endoscopic ultrasonography
MBCs: Mediastinal bronchogenic cysts
PBCs: Pulmonary bronchogenic cysts
SBCs: Spinal bronchogenic cysts
DBC: Diaphragmatic bronchogenic cyst
NBCs: Neck bronchogenic cysts
